# Who's Interested in Global Warming?

**DOI:** 10.1111/sjop.70007

**Published:** 2025-07-20

**Authors:** Mark Fenton‐O'Creevy, Adrian Furnham

**Affiliations:** ^1^ Open University Business School Milton Keynes UK; ^2^ Department of Leadership and Organisational Behaviour Norwegian Business School (BI) Olso Norway

**Keywords:** climate change, conspiracy theories, optimism, personality, political ideology

## Abstract

We report on a study of the correlates of attitude to global warming (GW). We build on prior research on the role of demographic variables, personality, and political orientation in predicting attitude to GW. We argue dispositional optimism should increase willingness to treat GW seriously, via its impact on active coping behaviors and reducing cognitive avoidance in the face of anxiety, and that there should be an interactive effect of optimism with political orientation. We draw on an existing data set (*N* = 819) of adult respondents. We use correlation and regression analysis to examine the association between demographic variables, personality traits, optimism, political orientation and GW attitude. We use moderated regression to test for an interactive effect between political orientation and optimism on GW attitude. We find a significant inverse association between (more right‐wing) political orientation and GW attitude, and a positive association between education and GW attitude. We find personality effects, the strongest of which is an inverse association between Competitiveness and GW attitude. As hypothesized, we find that optimism is positively associated with GW attitude and that this association is stronger for more right‐wing political orientation. We draw conclusions for the efficacy of approaches to communicating about climate change to different groups. We consider limitations of the research and implications for future research.

**Summary**

- We find a significant inverse association between (more right‐wing) political orientation and belief in the seriousness of global warming (GW attitude) and a positive association between education and GW attitude.
- There are personality effects, the strongest of which is an inverse association between Competitiveness and GW attitude.
- As hypothesised, optimism is positively associated with GW attitude and that this association is stronger for more right‐wing political orientation; hence optimistic communications about the potential for societal action to tackle global warming may have more influence with right‐wing audiences than fearful messages.

## 1 Introduction

There has been a huge increase in research on climate change attitudes (e.g., Christensen and Knezek 2015; Conversi and Friis Hau 2021; Dijkstra and Goedhart 2012; Douglas and Sutton 2015; Krange et al. 2018; van Valkengoed and Steg 2019; van der Linden 2016; Van Rensburg 2015). The global warming/climate change (GW) debate revolves around three issues: whether *unusual* climate change is actually occurring; the role of *human behavior* in those changes; and the extent to which we can *influence* the factors that affect GW (Dias et al. 2020). Climate skeptics argue that climate is, and has always been, changing due to natural, cyclical (non‐man‐made) forces. Their opponents, including most of the scientific community, argue that there is reliable evidence of dramatic change, with serious consequences, caused *primarily* by humans.

### 1.1 Climate Change Beliefs and Political Orientation

There has been some debate regarding the use of the terms “global warming” or “climate change” (Lineman et al. 2015; Schuldt et al. 2017). Schuldt et al. (2011) demonstrated that changing the question's wording from global warming to climate change resulted in a 6.3 percentage point increase in belief in environmental phenomena. They also found that this association was moderated by political self‐identification. Soutter and Mottus (2020) replicated this study in the USA, UK, and Australia and discovered that question wording no longer had a significant effect. Nonetheless, the association between political self‐identification and beliefs in environmental phenomena was replicated in all three countries. Conservatives consistently believed less in climate change/global warming than liberals (Soutter and Mottus 2020).

One explanation for the association of right‐wing beliefs with global warming denial lies in extensive research on epistemic motivations for political ideology. Jost et al. (2003) conducted a major integrative review of the correlates of right‐wing beliefs and attitudes. Drawing on theories of motivated social cognition, they identified the persistent association of a right‐wing orientation with (inter alia) the motivated desire to reduce uncertainty and avoid fear of threats.

Others have pointed to the role of conspiracy theories (an important component of global warming denial narratives) in restoring a sense of epistemological control in periods of unsettled insecurity (e.g., Carlson and Ramo 2023). Complementary research points to the association between right‐wing orientation and avoidant coping strategies (e.g., Kempthorne and Terrizzi Jr 2021). Thus, we hypothesize that (H1) there will be an inverse association between right‐wing beliefs and taking global warming seriously.

### 1.2 Climate Change Beliefs and Optimism

Dispositional optimism is a generalized tendency to expect positive outcomes (Carver et al. 2010) and is associated with active coping behaviors, responding positively and constructively to obstacles and setbacks (Conversano et al. 2010). Optimists are also less prone to cognitive avoidance when faced with anxiety‐provoking situations (Carver et al. 1999; Stanton and Snider 1993). Carver et al. theorize these relationships in terms of expectancy theory and motivated cognition, arguing that optimism increases the expectancy of goal success and leads to coping strategies that engage with causes of stress and the emotions they provoke, as opposed to motivated disengagement and avoidance through, for example, denial or avoidance of thinking about a troublesome issue and wishful thinking.

Given this association of optimism with lower avoidant coping and positive approaches to overcoming obstacles, we should expect people higher on optimism to be more likely to believe that the challenges of global warming can be surmounted and thus less likely to seek refuge in beliefs that global warming is either not happening or that nothing can be done. Hence, we hypothesize (H2) that optimism will be associated with taking global warming more seriously.

### 1.3 The Potential Role of Optimism in Ameliorating the Effects of Right‐Wing Orientation on Global Warming Attitudes

We have outlined above the evidence for an association between right wing orientation and avoidant coping strategies in the face of uncertainty and threat, and the role of optimism in buffering anxiety and reducing avoidant coping.

As we note above, there is significant evidence of the association between a right‐wing orientation and the motivated desire to reduce uncertainty and avoid fear of threats. In consequence, greater threat sensitivity will predispose political conservatives to reject arguments for climate change because of the threat it presents. Whereas less threat sensitivity and openness to social change among liberals will mean greater willingness to accept GW. Thus the resilience to threat and change offered by optimism may have a stronger effect for those more right‐wing than those more liberal, since it will reduce the perceived threat and uncertainty, reducing motivations for avoidant coping Carver et al. 1999, 2010).

Thus, we hypothesize (H3) that the inverse association between right wing beliefs and taking global warming seriously will be weaker for greater levels of optimism.

Our research question is focused on what individual difference factors are most consistently and powerfully associated with concerns about GW? We examine whether concerns about GW are related to demography (gender, age and education), political beliefs, optimism, and personality. Each has been implicated in previous research, and our question concerned the relative predictive power of each, in a large adult and international sample. We hypothesize, based on recent literature, that younger, better educated, politically liberal people, and people with a greater tolerance of ambiguity would be more concerned with GW (Leka and Furnham 2024). We further hypothesize an interactive effect between political orientation and optimism on global warming attitude, such that the relationship between optimism and GW concerns is stronger (weaker) for more right‐wing (liberal) political orientation.

In this study we considered the HPTI to be useful as unlike other similar Big‐Five‐type personality inventories it measures two additional variables not often assessed, namely Tolerance of Ambiguity and Risk Appetite, which we thought possibly related to GW. Moreover, it measures Competitiveness, the opposite of Agreeableness, which could also be relevant as it is associated with a desire for wealth, power, and success, and social dominance beliefs. While we make no explicit hypotheses concerning other personality factors than Tolerance of Ambiguity, we consider it important to include a wider range of personality variables in the analysis given mixed findings in prior research.

## 2 Methods

### 2.1 Participants and Procedure

We conducted a secondary analysis of an existing data set. Participants were recruited from a pool of individuals who had completed a psychometric assessment provided by the test publisher Thomas International for occupational test use, who subsequently volunteered to take part in psychology research, completing a further battery of questions. Participants were incentivized to take part by being offered brief feedback on their results following the study. Participants were emailed to inform them of the study and provide them with a link to information on the survey and the opportunity to give their informed consent for researchers to analyze and publish the anonymized data. The survey was conducted on an online survey platform. The research was approved by the ethics committee LSA/TI/2022. Participants were debriefed, thanked for their time, and provided feedback on their scores.

There were 819 respondents (472 females and 347 males), aged, on average, 46.07 years (ranging from 17 to 74; SD = 11.41). The largest national groups were British (426), South African (115), American (74), and Canadian (91) with the remainder from a mix of countries or not specifying nationality.

### 2.2 Variables

#### 2.2.1 Dependent Variable

Attitude to global warming: “How seriously do you take global warming?” responses on a 9‐point scale from 1 “not at all seriously”, to 9 “very seriously”. Mean (SD) = 6.65 (2.08).

#### 2.2.2 Independent Variables: Demographics

Age: In years. Mean (SD) = 46.06 (11.39).

Gender: 1 “female”, 0 “male”, (there were categories for “non‐binary” and “prefer not to say” but these were not used in the sample under consideration).

Have a degree: Whether educated to degree level 1 “yes”, 0 “no”.

#### 2.2.3 Independent Variables: Personality and Attitudes

*High Potential Trait Indicator (HPTI)* (MacRae and Furnham 2020). The HPTI is a measure of personality traits, specifically within a workplace context. The six traits are: *Conscientiousness*, characterized by self‐discipline, organization, and ability to moderate one's own impulses; *Adjustment* (low Neuroticism), characterized by emotional resilience to stressors, positive affect, and mood stability and regulation; *Curiosity* (Openness), characterized by an interest in new ideas, experiences, and situations; *Ambiguity Acceptance*, how individuals process and perceive unfamiliarity and uncertainty; *Competitiveness*, which is related to low Agreeableness and focuses on the drive for self‐improvement, desire for individual and team success, and learning; *Courage*, or *Approach to Risk*, which is the ability to combat or mitigate negative or threat‐based emotions and broaden the potential range of responses. The inventory is 78 items in length. It has been used in multiple studies (Cuppello et al.2023a, 2023b; Furnham and Treglown 2018; Teodorescu et al. 2017).

Optimism: Single item measure, participants were asked to characterize their personal optimism on a scale from 1 “not at all” to 9 “very”.

Political orientation: Single item measure, participants were asked to characterize their political views on a scale from 1 “very conservative” to 9 “very liberal”.

#### 2.2.4 Analysis

We first calculated Pearson correlations between all variables. Table 1 shows Pearson correlations and descriptive statistics (on the diagonal). Next, we conducted a hierarchical regression analysis (Table 2) in three steps: first regressing attitude to global warming on demographic variables, then adding the six HPTI factors. Next, we added political orientation and optimism. In a final step, we carried out moderated regression by adding an interactive term between political orientation and optimism. To aid interpretation of parameters, given the inclusion of an interactive term, we mean centered all independent variables. The descriptive statistics in Table 1 refer to the uncentered variables. The mean‐centered variables all have zero means but the same standard deviation as the uncentered variables. Variance inflation factors (VIFs) were checked for models 1 to 3; the largest (2.23 for risk approach in model 3) was well below the commonly accepted threshold of concern of 5 (Chatterjee and Simonoff 2013). VIFs were not checked for the final model with the interaction term as there is multicollinearity by design when entering an interaction term, but this poses no problems for interpretation (Hayes 2013).

**TABLE 1:** Correlations and descriptives.

	1	2	3	4	5	6	7	8	9	10	11	12
1. Global warming attitude	6.65 (2.08)											
2. Age (years)	0.05	46.07 (11.41)										
3. Gender (female)	0.05	−0.03	0.58 (0.49)									
4. Has degree	0.09^a^	−0.11^b^	0.08^a^	0.69 (0.46)								
5. Conscientiousness	0.01	0.11^b^	0.01	−0.04	54.60 (14.07)							
6. Adjustment	0.07^a^	0.22^b^	−0.08^a^	−0.01	0.23^b^	50.64 (16.25)						
7. Curiosity	0.10^b^	0.04	−0.05	0.09^b^	0.30^b^	0.21^b^	57.04 (13.63)					
8. Risk approach	0.02	0.21^b^	−0.15^b^	−0.03	0.50^b^	0.52^b^	0.45^b^	52.06 (13.67)				
9. Ambiguity acceptance	0.11^b^	0.23^b^	−0.06	0.05	0.17^b^	0.48^b^	0.31^b^	0.47^b^	46.11 (12.49)			
10. Competitiveness	−0.14^b^	−0.18^b^	−0.11^b^	0.01	0.35^b^	−0.02	0.06	0.23^b^	0.04	43.41 (14.62)		
11. Political orientation	0.25^b^	−0.09^a^	0.19^b^	0.16^b^	−0.06	−0.03	0.13^b^	−0.07^a^	0.06	−0.16^b^	5.48 (1.95)	
12. Optimism	0.12^b^	0.18^b^	0.08^a^	−0.02	0.22^b^	0.42^b^	0.28^b^	0.34^b^	0.25^b^	0.01	0.04	6.82 (1.88)

*Note:* Pearson correlations. Means (standard deviations) on the diagonal. *N* = 819.

^a^ Correlation is significant at the 0.05 level (2‐tailed).

^b^ Correlation is significant at the 0.01 level (2‐tailed).

**TABLE 2:** Hierarchical regression predicting global warming attitudes.

Coefficients^a^	Model															
	1				2				3				4			
	*B*	*β*	*t*	Sig.	*B*	*β*	*t*	Sig.	*B*	*β*	*t*	Sig.	*B*	*β*	*t*	Sig.
(Constant)	6.65		91.76	< 0.001	6.65		93.160	< 0.001	6.65		95.58	< 0.001	6.66		95.85	< 0.001
Age (years)^b^	0.01	0.06	1.71	0.088	0.00	0.01	0.19	0.850	0.00	0.02	0.59	0.558	0.01	0.03	0.76	0.445
Gender (female)^b^	0.19	0.04	1.26	0.208	0.12	0.03	0.82	0.414	−0.07	−0.02	−0.47	0.637	−0.07	−0.02	−0.45	0.655
Has degree^b^	0.42	0.09	2.64	0.009	0.36	0.08	2.26	0.024	0.25	0.06	1.63	0.105	0.24	0.05	1.53	0.127
Conscientiousness^b^					0.01	0.05	1.13	0.258	0.01	0.04	1.04	0.299	0.01	0.05	1.18	0.240
Adjustment^b^					0.01	0.04	0.83	0.410	0.00	0.01	0.30	0.766	0.00	0.01	0.28	0.777
Curiosity^b^					0.01	0.08	2.00	0.046	0.00	0.03	0.73	0.467	0.01	0.03	0.82	0.413
Risk approach					−0.01	−0.06	−1.25	0.214	−0.01	−0.05	−1.00	0.317	−0.01	−0.05	−1.05	0.294
Ambiguity acceptance^b^					0.02	0.09	2.12	0.035	0.01	0.07	1.67	0.096	0.01	0.07	1.73	0.085
Competitiveness^b^					−0.02	−0.15	−3.89	< 0.001	−0.02	−0.12	−3.05	0.002	−0.02	−0.11	−2.98	0.003
Political orientation (PO)^b^									0.23	0.22	6.16	< 0.001	0.24		6.34	< 0.001
Optimism (Opt)^b^									0.10	0.09	2.38	0.017	0.10		2.26	0.024
PO^b^ × Opt^b^													−0.04		−2.29	0.022
Model *R* ^2^ (Adj.*R* ^2^)	0.01 (0.01)		0.012		0.05 (0.04)			< 0.001	0.10 (0.09)			< 0.001	0.11 (0.09)			< 0.001
*R* ^2^ change					0.04			< 0.001	0.05			< 0.001	0.01			0.022

*Note:* Standardized regression coefficients are not reported for terms in the interaction in model 4, as they are misleading. *N* = 819.

Abbreviations: *β*, standardized regression coefficients; *B*, unstandardised regression coefficients.

^a^ Dependent Variable: Attitude to global warming.

^b^ Mean centred.

## 3 Findings

Table 1 gives Pearson correlations and descriptive statistics. Six variables were significantly related to how seriously the participants took GW. Graduates had higher scores than non‐graduates; politically liberal more than conservative people; and those who were more optimistic. Four traits were implicated: Adjustment, Ambiguity Acceptance, and Curiosity positively related to taking GW seriously, and Competitiveness negatively.

Table 2 shows the results of the hierarchical regression and moderated regression.

The first step included the three demographic variables and suggested graduates were more concerned about GW than non‐graduates. The second step added the six traits and showed three were significant in addition to the graduate status, of which the most important was Competitiveness. The third step added political orientation and optimism. This showed the combined factors accounted for a tenth of the variance: Politically liberal people, those who self‐rated as more optimistic, and those who were less Competitive were more concerned about GW. Curiosity and Ambiguity Acceptance were significant in model 2 but fell below the 0.05 significance threshold in model three, suggesting their relationships with GW attitude may be mediated via the final variables to be entered.

Model 4 shows the addition of the interaction term. The significant (at *p* < 0.05) interaction parameter and change in *R* ^2^ support the hypothesized interactive effect on GW attitude. Note, we do not include standardized regression coefficients for the variables in the interaction since these can be misleading in moderated regression (since the *z*‐score of the product does not equal the product of *z*‐scores and the effect size of one variable is conditioned on another).

**FIGURE 1:**
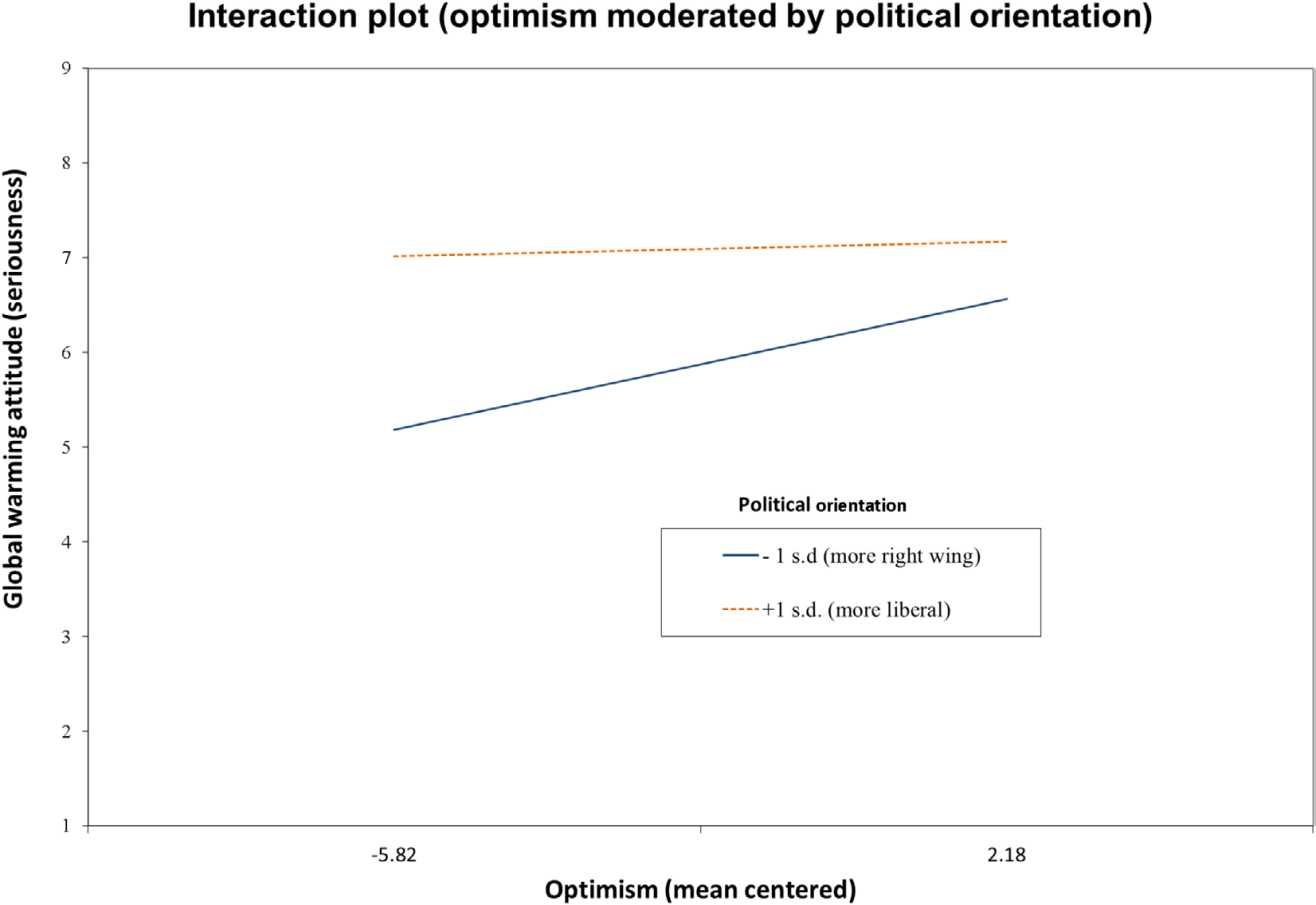
Interaction of political orientation and optimism predicting global warming attitude.

To aid interpretation of the interactive effect, we chart the relationship between optimism and GW attitude for political orientation scores one standard deviation either side of the mean (Figure 1). Results are shown across the range of observed values for optimism (with other variables held at their mean values). We see that, in line with the hypothesis, the relationship between optimism and GW attitude is stronger for more right‐wing orientation than for more liberal orientation.

Simple slopes tests show that at −1 SD, the slope gradient (0.17) is significant (*t* = 3.26, *p* < 0.001), whereas at +1 SD, the slope gradient (0.02) is not significantly different from zero (*t* = 0.36, *p* = 0.722). We also conducted simple slopes tests for the midpoint of the scale (5 in the uncentred version) and for 1 point above the scale centre (6 in the uncentred version), since these demark the transition from more right‐wing to more liberal orientation. At the midpoint, the gradient of the slope (0.12) is significant (*t* = 2.80, *p* = 0.005) but the gradient (0.08) becomes non‐significant at 1 point above the scale centre. The gradient remains non‐significant at the highest (most liberal) point of the scale.

Whilst standardized regression coefficients for terms in an interaction are not readily interpretable, since effect size is conditioned on the moderator, we can manually calculate an equivalent at different levels of political orientation. We calculated the effect of a 1 standard deviation increase in optimism on global warming attitude for different levels of political orientation using the regression equation implied by the parameters in model 4, holding all other variables at their mean level (zero in their centred forms). We express the differences in standard deviation units. For a value of political orientation 1 standard deviation below the mean (right wing),1 standard deviation increase in optimism is associated with a 0.16 standard deviation increase in global warming attitude. For political orientation 1 standard deviation above the mean (liberal) 1 standard deviation increase in optimism is associated with just a 0.04 standard deviation increase in global warming attitude.

Thus, the results of model 4 suggest that optimism has a significant positive relationship with GW attitude for those with a right‐wing or neutral political orientation, but not for those with a liberal political orientation, and the strength of the relationship increases with the strength of right‐wing orientation.

## 4 Discussion

This study highlighted the dominance of political beliefs in relation to attitude to climate global warming. Public discussions of GW have been heavily politicized, so much so that attitudes and beliefs seem to be dominated by politics. This presents a particular challenge in attempting to educate people or change their beliefs about GW. Graduates took GW more seriously than non‐graduates, and the effect of education may have been stronger had we had a greater range of participants.

Whilst we made no hypothesis about Competitiveness, this study also highlighted the role of trait *Competitiveness*, which is related to low Agreeableness and focuses on the drive for self‐improvement, desire for individual and team success, and learning. The manual of the HPTI notes that high scorers thrive and are motivated by competitive environments and turn most activities into competitions. They want to be the winner, acknowledged and rewarded as such, and prefer to be in a position of power, authority, or influence. Competition seems to drive you to higher levels of performance and can make even ordinary tasks more enjoyable or engaging. We also note the association between a competitive worldview and social dominance orientation (Osborne et al. 2023). Social dominance orientation has been found to predict climate change denial as a consequence of the motivation to protect social as well as human‐nature hierarchies, since social domination orientation concerns the desire to protect the privileges of dominant groups (Jylhä and Akrami 2015). It is less privileged groups that face the most severe consequences of global warming whilst the urgent need for behavioral change falls mostly on more privileged groups. We cannot over‐rely on findings about Competitiveness in relation to a single study that did not make a prior hypothesis. However, it does raise the question as to whether those higher in competitiveness may be more readily influenced by global warming messaging that emphasizes the impacts on those like themselves rather than relying on empathy for disadvantaged groups.

It was notable in the second regression model that two other traits reached significance. Curiosity or Openness was positively associated with a concern about GW. Openness is correlated with intelligence and the need for understanding which explains why they may be more interested in and concerned about the GW debate. Similarly, Ambiguity Acceptance is related to concerns about GW. This finding is similar to that of Alfasi (2023) who found Intolerance of Ambiguity associated with conspiracy theories, particularly the COVID issue. The literature on Ambiguity Acceptance goes back over 70 years and it has consistently been inversely associated with authoritarianism and conservatism. Low tolerance of epistemic uncertainty is associated with more right‐wing thinking and the acceptance of a range of conspiracy theories.

In recent years, the terms “deniers”, “contrarians”, “dissenters”, and “skeptics” have been used interchangeably to describe the portion of the public that take scientific uncertainty as an absence of consensus on climate change, or who publicly misrepresent climate science and attack scientific claims: usually described as climate conspiracists (McCright 2007). This is in line with Tobler et al. (2012) and Malpass et al. (2007) who argue that climate skeptics tend to distrust scientific facts, harbor doubts about individual responsibility for climate change and endorse other conspiracy theories.

We considered the effect of optimism. There is evidence that people are less inclined to believe things that make them fearful (Kaplan et al. 2016). Thus optimists, more likely to believe problems are surmountable, may be less resistant to narratives about the seriousness of GW, because they make them less fearful. We also found support for the greater importance of optimism to accepting the seriousness of GW for those with right‐wing than for those with liberal political orientation. As we argued in developing the hypothesis, this may well be because of the association of right‐wing orientation with avoidant coping when confronted with uncertainty and threat, and the importance of optimism in buffering anxious responses to uncertainty and threat and reduced avoidant coping.

Understanding the relationship between GW beliefs, personality, optimism, and political beliefs offers the opportunity to craft communications about GW that may more effectively target different audiences. In particular, our analysis suggests that fearful messages about climate change may be especially unhelpful for more right‐wing audiences as they may exacerbate the tendency for threat sensitivity to generate avoidant coping via motivated cognition, such as resorting to conspiracy theories to escape threat and uncertainty. Rather, messaging that promotes optimism about the potential for societal action to overcome the threat of global warming may be most helpful in addressing these audiences.

## 5 Limitations

One obvious limitation is that several variables were measured by a single item. However, there is now increasing evidence that single item measures are both robust and valid. An editorial in the *European Journal of Psychological Assessment* (Volume 38:1) was dedicated to this issue and the editors concluded “most research published on single‐item measures shows that they are often as valid and reliable as their multi‐item counterparts” (Allen et al. 2022, 4). Further, single item measures have the merit of reducing demands on research participant time and thus reduce problems of inattention that often arise in lengthy surveys.

A particular criticism leveled at single item measures is that they are more prone to random error. This can reduce the power of tests. In this study, this is mitigated by the relatively large sample. However, the most likely main effect of random error would be to attenuate the size of parameters in tests of association such as regression and correlation, meaning that real effect sizes may be greater than detected.

Nonetheless, richer measures of constructs can pick up much greater nuance. It would, for example, have been useful to have items on global warming that examine different facets of the seriousness with which participants take global warming; for example, belief in the evidence that human‐caused global warming is happening, the seriousness of the likely effects, the impacts on human and other life, and the potential for action to reduce global warming.

A second limitation concerns the modest variance explained in our analysis. This may in part be due to attenuation of parameters due to possibly higher random error in the single item measures. However, it also suggests that important sources of variance in attitudes to global warming remain unmeasured. Key additional variables that may be useful to measure in future work could include trust in science and exposure to, and belief in, conspiracy theories. The “consideration of future consequences” scale (the extent to which people consider distant versus immediate consequences of potential behaviors) would also be a construct with the potential to explain significant variance in climate attitudes (Beiser‐McGrath and Huber 2018).

It would have been most interesting to know more about the participants' knowledge and interest in GW as well as how it influences their lifestyle and voting pattern. Similarly, it would be desirable to have a deeper understanding of participants' political beliefs and behaviors. Nonetheless, within the limitations of the data on which we conducted secondary analysis, the findings offer an intriguing perspective on how different audiences might be more effectively addressed in communications about the importance of action to address global warming.

## Author Contributions

M.F.‐O.: data analysis; writing, review and editing. A.F.: data curation: writing review and editing.

## Ethics Statement

This was sought and obtained (SLA/TI/2022).

## Consent

Participants gave consent for their anonymised data to be analyzed and published.

## Conflicts of Interest

The authors declare no conflicts of interest.

## Data Availability

The data set on which we rely is copyright of Thomas International and cannot be deposited in a public repository. However, the subset of data analysed for this paper may be requested from the corresponding author.
